# The Effect of Seasonal and Annual Variation on the Quality of *Polygonatum Cyrtonema* Hua Rhizomes

**DOI:** 10.3390/plants13243459

**Published:** 2024-12-10

**Authors:** Weiting Pu, Yefei Yu, Xiaoxiao Shi, Ye Shao, Bihuan Ye, Youwu Chen, Qiyan Song, Jianjun Shen, Haibo Li

**Affiliations:** 1 School of Forestry and Biotechnology, Zhejiang Agriculture and Forestry University, Hangzhou 311300, China; weitingpu@163.com (W.P.);; 2 Zhejiang Dapanshan National Natural Reserve Administration, Panan 322300, China; feieryun1126@126.com; 3 Zhejiang Academy of Forestry, Hangzhou 310023, China

**Keywords:** *Polygonatum cyrtonema*, medicinal plant, growth period, quality, functional components

## Abstract

This study aims to reveal the interannual and seasonal variations in functional components in *Polygonatum cyrtonema* Hua. rhizomes and evaluate whether the variations significantly affect the quality of rhizomes as a traditional Chinese herbal medicine. The interannual and seasonal variations in total flavonoid content and total saponin content were analyzed. The global dynamic variation in secondary metabolites in the rhizomes during a five-year growth period and in two traditional harvesting seasons were investigated based on metabolomics method. Results clearly showed that the functional components in *P. cyrtonema* rhizomes exhibited a significant increase in accumulation during the one- to four-year growth period and a significant decrease in accumulation during the four- to five-year growth period. The most active accumulation occurred during the three- to four-year growth period. Drastic variations in functional components occurred from spring to autumn. The significant interannual variation and drastic seasonal variation were strongly associated with the enrichment in some pathways related to the biosynthesis of secondary metabolites and the metabolisms of amino acids. The interannual and seasonal variations in functional components significantly affected the quality of *P. cyrtonema* rhizomes. The four-year-old rhizomes had the most superior quality due to their higher content of functional components and much more newly formed components. The rhizomes harvested in spring or autumn had unequal quality because of their significant differences in composition and content of functional components. Specifically, the rhizomes from spring contained more flavonoids, alkaloids, and phenolic acids, while those from autumn comprised more steroids. In conclusion, this study reveals that the interannual and seasonal variations in functional components can significantly affect the quality of *P. cyrtonema* rhizomes as a traditional Chinese herbal medicine. This study provides foundational insights and theoretical guidance for determining an optimal cultivation period to obtain medicinal rhizomes with superior quality. It also offers a strategy for harvesting medicinal rhizomes in two different seasons to achieve unequal quality.

## 1. Introduction

Species in the genus *Polygonatum* are perennial herbs of Asparagaceae and widely distributed in China. The underlying pharmacological applications of *Polygonatum* are gaining popularity in clinical diseases, such as fatty liver disease, Alzheimer’s disease, diabetes mellitus, and cancer [1]. Recently, *Polygonatum* spp. have become widely used in Traditional Chinese Medicines (TCMs) in improving diabetes [2]. Such biological activities are attributed to the abundant functional medicinal components of *Polygonatum* derived from its rhizomes, including polysaccharides and secondary metabolites (e.g., saponins, flavonoids, phenolics, alkaloids, and lignans) [2,3]. Among flavonoids, homoisoflavones, which are a small, rare, and unique class of flavonoids, are characteristic components of *Polygonatum* plants with antimutagenic, antioxidant, immunomodulating, antidiabetic, cytotoxic, antiangiogenic, and vasorelaxant medicinal efficacy; thus, they can be used as potential clinical medicines [4].

The species *P. cyrtonema* Hua. (“Duohua Huangjin” in Chinese), which is a known medicinal plant, is recorded in China Pharmacopoeia and has a long history of application in Chinese folk medicine [5,6]. The quality of *P. cyrtonema* rhizomes depends on the composition and content of functional components contained in them, which directly affect their clinical efficacy as a TCM. These extrinsic factors including cultivation environment, cultivation stage, and harvesting period can significantly influence the chemical compositions and content, which determine the quality of rhizomes. Some evidence supported that the functional components in TCMs are constantly changing in different growing periods, as shown by the study on *Scutellariae radix* and *Angelica sinensis.* The growth years can greatly affect the content of four key active components, namely, baicalin, wogonoside, baicalein, and wogonin in *S. radix*. The total content of the four components in two-year-old *S. radix* roots was the highest, followed by the three- and four-year-old roots, with the lowest in one-year-old roots. During the entire growth cycle of *A*. *sinensis*, more changes in active constituents including ferulic acid, ligustilide, and angelica polysaccharide occurred during the two-year and three-year growing period, and the changes in angelica polysaccharide were greater than in erulic acid and ligustilide; as a result, the harvested roots or rhizomes exhibit significant differences in quality and clinical efficacy [7]. The *P. cyrtonema*, as a famous Chinese folk herbal medicine, is usually cultivated for five years and harvested in spring or autumn [8]. The entire rhizome of five-year-old *P. cyrtonema* consists of five age sections including one-, two-, three-, four-, and five-year-old age sections. Initial studies provided primary evidence for the proper harvesting time for rhizomes. They compared differences in polysaccharides, extractives, and functional components across age sections during a three-year growth period and between different harvesting seasons; the studies revealed that cultivation years and harvesting periods directly impacted the quality and effectiveness of *P. cyrtonema* rhizomes as Chinese medicinal materials [8,9,10,11]. However, *P. cyrtonema* is usually cultivated for five years in producing areas in China. Therefore, a three-year growth period is insufficiently long to study its interannual variation in functional components. Moreover, a comparative analysis based on only polysaccharides and alcohol-soluble extractives cannot accurately reflect the global variation in functional components of *P. cyrtonema* rhizomes. Considering the lack of a comprehensive investigation into the metabolic composition of *P. cyrtonema* rhizomes during a five-year growth period, the interannual and seasonal variations in functional components and whether the variations significantly affect the quality of rhizomes as a traditional Chinese herbal medicine still remain unclear. In addition, whether spring or autumn was traditionally considered the optimal harvesting period for obtaining high-quality rhizomes is doubtful. Ensuring their efficacy also lacks sufficient evidence to date.

Metabolomic research has emerged as a valuable technology for the comprehensive profiling and comparison of metabolites as end products of cellular regulatory processes; their levels can be regarded as the ultimate response of biological systems to genetic or environmental changes [12]. Based on the nonbiased and high-throughput analyses of complex metabolites contained in plant extracts by HPLC-MS (for nonvolatile compounds), GC-MS (for volatile oil), or NMR, plant metabolomics has been gradually applied to the studies on planting and cultivation, harvesting and processing, and identification and analysis of rhizomes of TCM; this approach provided a certain data basis for the dynamic study on the time distribution of active ingredients in medicinal parts and for the selection of growing years [7,13]. Our study focuses on the functional components derived from *P. cyrtonema* rhizomes. Specifically, metabolomic analysis was performed to investigate the dynamic changes in *P. cyrtonema* rhizomes during a five-year growth period. Our objectives were to reveal the interannual and seasonal variation trends of functional components in *P. cyrtonema* rhizomes and evaluate whether the variations significantly affect the quality of rhizomes as a traditional Chinese herbal medicine.

## 2. Results

### 2.1. Interannual and Seasonal Variations in Total Flavonoid Content and Total Saponin Content in P. cyrtonema Rhizomes

**Figure 1:**
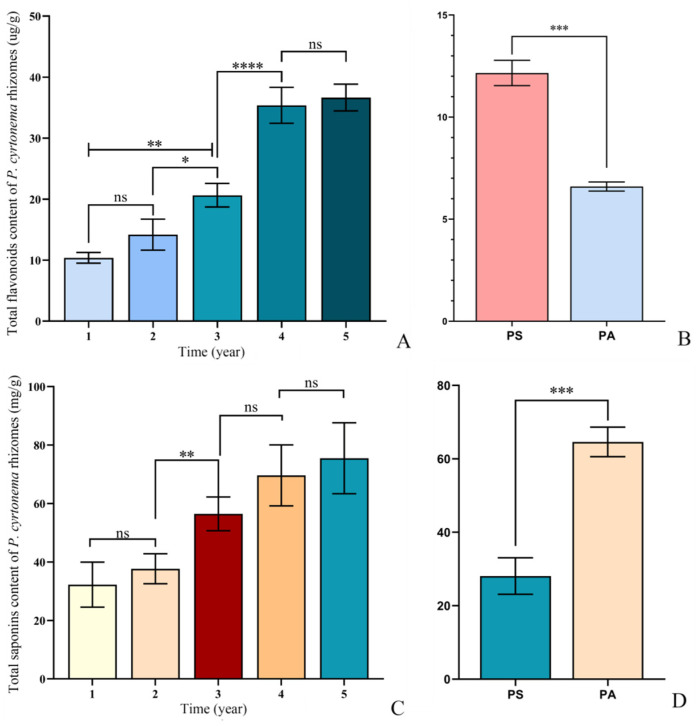
Total flavonoid content and total saponin content in *P. cyrtonema* rhizomes. (**A**), Total flavonoid content in five age sections; (**B**), total flavonoid content in entire rhizomes in spring and autumn; (**C**), total saponin content in five age sections; (**D**), total saponins content in entire rhizomes in spring and autumn. The letters PS and PA refer to the rhizomes harvested in spring and autumn, respectively. The abbreviated ns represents no significant difference. ****, ***, **, and * represent statistically significant at *p* < 0.0001, *p* < 0.001, *p* < 0.01, and *p* < 0.05, respectively.

With regard to the interannual variation in total flavonoids, their content in the five age sections of five-year-old *P. cyrtonema* rhizome ranged from 9.69 μg/g to 35.39 μg/g, and they showed a gradually increasing trend with the progressing growth years in general (Figure 1A). Among them, the content in the three-year-old age sections was significantly higher than that in the one- and two-year-old age sections (*p* < 0.01 and *p* < 0.05). The content in the four-year-old age sections was significantly higher than that in the three-year-old age sections (*p* < 0.0001). The content between the one- and two-year-old age sections, as well as between the four- and five-year-old age sections, exhibited insignificant differences. In addition, the total flavonoid content in the entire rhizomes harvested in spring was significantly higher than that in autumn (*p* < 0.001), which showed a seasonal decrease in total flavonoid content (Figure 1B).

For the interannual variation in total saponins, their content in the five age sections of five-year-old *P. cyrtonema* rhizome ranged from 21.15 mg/g to 90.54 mg/g, and they showed a gradually increasing trend with the progressing growth years in general (Figure 1C). Among them, the content in the three-year-old age sections was significantly higher than that in the one- and two-year-old age sections (*p* < 0.01). The content between the one- and two-year-old age sections, between the three- and four-year-old age sections, and between the four- and five-year-old age sections showed insignificant differences. In addition, the total saponin content in the entire rhizomes harvested in spring was significantly lower than that harvested in autumn (*p* < 0.001), which showed a seasonal increase in total saponin content (Figure 1D). These results reflect the significant interannual and seasonal variations in two key functional components including flavonoids and saponins during the five-year growth period and in the two harvesting seasons, which suggest that the variations in functional components significantly affected the quality of *P. cyrtonema* rhizomes as a Chinese herbal medicine.

### 2.2. Global Metabolomic Profile of P. cyrtonema Rhizomes

**Figure 2:**
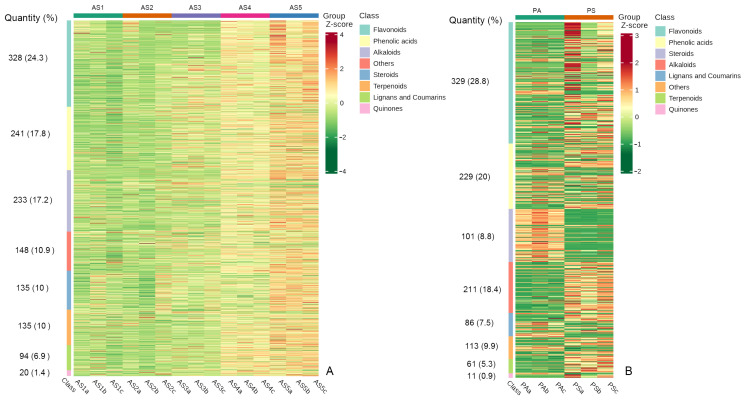
Basic information on secondary metabolites detected from *P. cyrtonema* rhizomes. (**A**), Clustered heatmap of secondary metabolites in samples of one-year-old age sections (AS1 group), two-year-old age sections (AS2 group), three-year-old age sections (AS3 group), four-year-old age sections (AS4 group), and five-year-old age sections (AS5 group); (**B**), clustered heatmap of secondary metabolites in samples of spring rhizome (PS group) and autumn rhizome (PA group). The shades of color indicate the quantity of metabolites, with redder shades representing more and greener shades representing fewer.

Based on the UPLC-MS/MS detection platform and the Metware database, secondary metabolites in five age sections (AS1, AS2, AS3, AS4, and AS5 groups) of five-year-old *P. cyrtonema* rhizome and in two seasonal rhizomes (PS and PA groups) of five-year-old *P. cyrtonema* were determined. A total of 1349 metabolites were tentatively identified from the five age sections and 1,141 metabolites from the two seasonal rhizomes. The main secondary metabolites in the age sections of five-year-old *P. cyrtonema* rhizomes were flavonoids, phenolic acids, and alkaloids, which accounted for 59.5% of the total; they were followed by steroids and terpenoids, which accounted for 20% of the total (Figure 2A). The main secondary metabolites in the seasonal rhizomes were flavonoids, phenolic acids, and alkaloids, which accounted for 57.2% of the total; they were followed by steroids, lignans and coumarins, and terpenoids, which accounted for 21.6% of the total (Figure 2B). Meanwhile, the hierarchical clustering heatmap based on UV scaling showed that the relative abundance of most secondary metabolites in the one-, two-, and three-year-old age sections exhibited insignificant differences. However, most secondary metabolites in the four-year-old age section (AS4) were obviously more abundant than those in the three-year-old age section (AS3 group). Those in the five-year-old age section (AS5) were slightly more abundant than those in the four-year-old age section (AS4 group). For the seasonal rhizomes, most secondary metabolites, except steroids in the spring rhizomes (PS group), were obviously more abundant than those in the autumn rhizomes (PA group). These results initially indicate that the interannual variation in secondary metabolites occurred during the five-year growth period, and the seasonal variation occurred during the spring–autumn growth period.

The differences in overall metabolites among the five interannual groups and between the two interannual groups were determined, and the extent of variability between rhizome samples within the same group was investigated. For these purposes, UV scaling and PCA were performed on fifteen interannual samples and six seasonal samples, respectively. For the interannual groups, the explained variance ratios of PC1 and PC2 in the PCA plot were 48.97% and 14.03%, respectively (Figure S1A). For the seasonal groups, the explained variance ratios of PC1 and PC2 in the PCA plot were 55.57% and 11.94%, respectively (Figure S1B). Three mixed samples as QC were grouped together near the center of the PCA plot, which ensured that QC samples had similar metabolic profiles and guaranteed the stability and repeatability of the entire analysis. The PCA plot showed that three replicates within the AS1 group, three replicates within the AS2 group, and three replicates within the AS3 group were not clearly separated; instead, the nine samples nearly clustered into a large group. Moreover, three replicates within the AS4 group were completely separated from the nine samples from the AS1, AS2, and AS3 groups. They were also separated from three replicates within the AS5 group in the PCA plot. The initial finding from PCA plots suggested that the secondary metabolites in the one-, two-, and three-year-old age sections showed no remarkable differences. However, the secondary metabolites in the four-year-old age sections exhibited remarkable differences from those in the one-, two-, and three-year-old age sections. These metabolites also had obvious differences from those in the five-year-old age sections. In addition, the PCA plots showed that the three replicates within the PS group were completely separated from the three replicates within the PA group, which suggests that the secondary metabolites in the entire rhizomes harvested in spring were significantly different from those harvested in autumn.

### 2.3. Differential Accumulated Secondary Metabolite Profiling of P. cyrtonema Rhizomes

PCA is typically insensitive to variables with small correlations. Thus, OPLS-DA multivariate statistical analysis was performed to generate the score plot of each group based on the differential variables, which revealed the separation among *P. cyrtonema* rhizome groups and identified significantly different secondary metabolites between these groups. The OPLS-DA score plots for the pairwise comparisons of AS2 vs. AS1, AS3 vs. AS2, AS4 vs. AS3, AS5 vs. AS4, and PA vs. PS, are shown in Figure S2. In the five OPLS-DA models, the values of R^2^Y and Q^2^ for the comparison groups were closer to 1.0, except for the comparison of AS2 vs. AS1 (Q^2^ = 0.849). This result indicates that these models were reliable and suitable for the following detection of differential metabolites.

**Figure 3:**
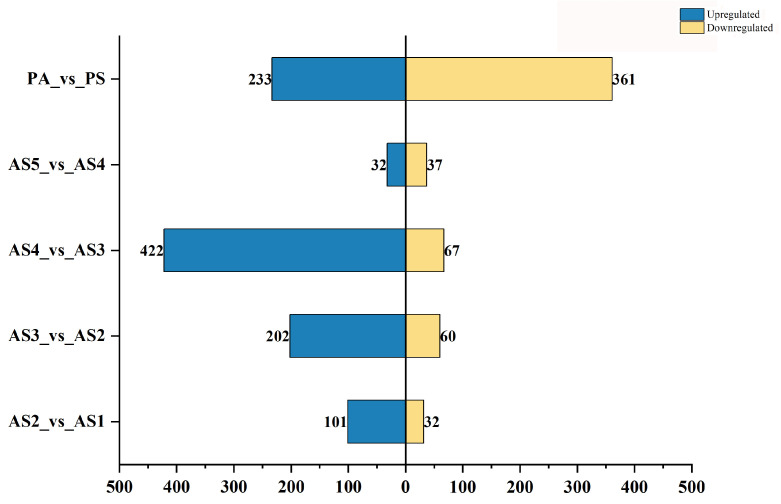
Quantity of differential accumulated secondary metabolites (DASMs) in five pairwise comparisons.

**Figure 4:**
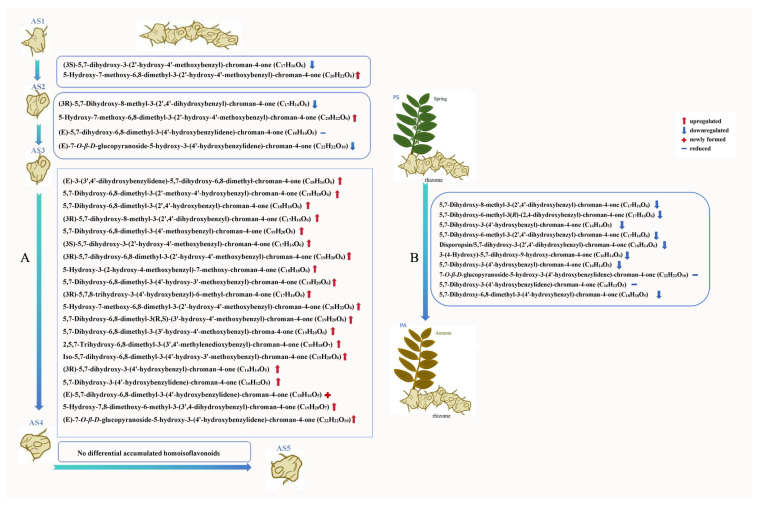
The differential accumulated homoisoflavonoids in *P. cyrtonema* rhizomes. (**A**), In five age sections of rhizomes during the entire five-year growing period; (**B**), in entire rhizomes during the growing period from spring to autumn. The letters AS1, AS2, AS3, AS4, and AS5 refer to one-, two-, three-, four-, and five-year-old age sections, respectively.

The significantly different secondary metabolites were considered as differential accumulated secondary metabolites (DASMs) within each pair of comparison groups based on fold change ≥ 2.0 [log_2_ (2) ≥ 1.0] or ≤0.5 [log_2_ (0.5) ≤ − 1] and VIP ≥ 1.0 as the screening standards. A total of 133 DASMs (101 upregulated and 32 downregulated) were detected from AS2 vs. AS1, 262 DASMs (202 upregulated and 60 downregulated) from AS3 vs. AS2, 489 DASMs (422 upregulated and 67 downregulated) from AS4 vs. AS3, 43 DASMs (32 upregulated and 11 downregulated) from AS5 vs. AS4, and 594 DASMs (233 upregulated and 361 downregulated) from PA vs. PS (Figure 3). The detailed information on the top 20 DASMs with maximum values of log_2_ (fold change) in each pair of comparison groups is listed in Table 1. Figure 3 shows that most DASMs detected from the interannual pairwise comparisons of AS2 vs. AS1, AS3 vs. AS2, and AS4 vs. AS3 were upregulated. Meanwhile, nearly equal quantities of up- and downregulated DASMs (32 and 37) were detected from the comparison of AS5 vs. AS4. However, in the seasonal comparison of PA vs. PS, much more downregulated DASMs (361) were detected than upregulated DASMs (233). In addition, a total of 31 flavonoids in the flavonoids class were identified as homoisoflavonoids. Among them, 20 were upregulated in the comparison of AS4 vs. AS3; no differential accumulated homoisoflavonoids were found in the comparison of AS5 vs. AS4; 10 were downregulated in the comparison of PA vs. PS (Figure 4). The detailed information on differential accumulated flavonoids and steroids in the five comparison groups is listed in Table 2.

In the pairwise comparison groups, except the DASMs with changed relative abundance, some DASMs were only detected in one group, which indicates that they were newly formed metabolites or reduced metabolites. A total of 12 and 31 newly formed metabolites were found in the pairwise comparison of AS3 vs. AS2 and AS4 vs. AS3, respectively, which constituted most of all newly formed metabolites in all four interannual comparison groups; only small quantities of reduced metabolites were detected in each pair of comparison groups (Table 3). In addition, a large quantity of newly formed metabolites (122) and reduced metabolites (148) were found in the seasonal comparison of PA vs. PS. The detailed information on the newly formed and reduced metabolites in each comparison groups is listed in Table S1.

These results indicate that the secondary metabolites in the one to four-year age sections of *P. cyrtonema* rhizomes had a significant increase in accumulation. The most abundant secondary metabolites were accumulated in the four-year age sections. The secondary metabolites had a significant decrease in accumulation in the four- to five-year age sections. In addition, these results indicate that drastic changes in types and abundance of functional components in rhizomes occurred during the spring–autumn growth period. These findings suggest that the interannual variation in functional components during the five-year growth period and their drastic seasonal variation during two harvesting seasons significantly affect the quality of rhizomes as a traditional Chinese herbal medicine.

### 2.4. Interannual and Seasonal Variations in Secondary Metabolites from P. cyrtonema Rhizomes

**Figure 5:**
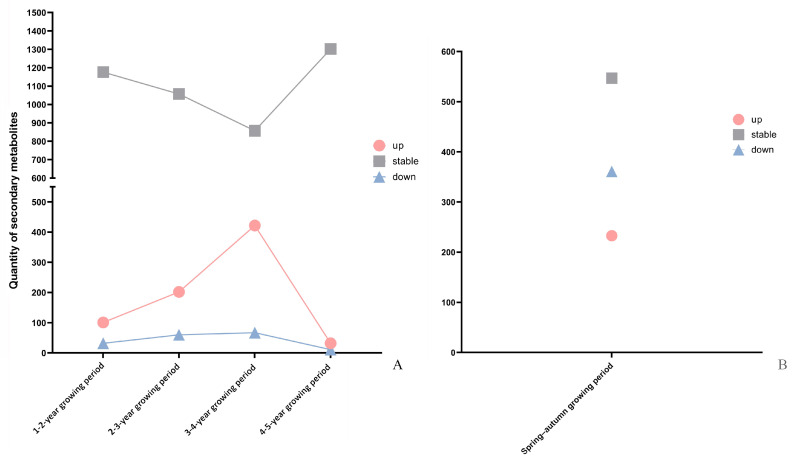
Interannual and seasonal variation in secondary metabolites in *P. cyrtonema* rhizomes. (**A**), From the one- to five-year growing periods; (**B**), during the spring–autumn growing period.

**Figure 6:**
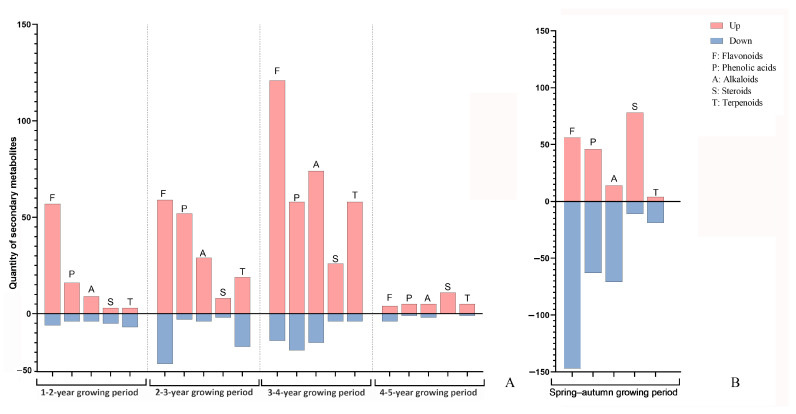
Class and quantity of main differential accumulated secondary metabolites (DASMs) in *P. cyrtonema* rhizomes. (**A**), From the one- to five-year growing periods; (**B**), during the spring–autumn growing period.

A line graph and a scatter plot were created based on the changes in composition and abundance of secondary metabolites in *P. cyrtonema* rhizomes, respectively. They were established to further describe the interannual variation in these rhizomes during the entire five-year growth period and their seasonal variation from spring to autumn (Figure 5A,B). The upregulated DASMs in the interannual rhizome samples exhibited a continuously increasing trend from the first year; then, they reached a maximum quantity of 422 (31.35%) by the fourth year; subsequently they decreased to a minimum quantity of 32 (2.38%) by the fifth year (Figure 5A). Among them, the most significantly increased compounds were flavonoids; they were followed by phenolic acids, alkaloids, and terpenoids; steroids were the least increased compounds (Figure 6A). Compared with the variation in the abundant upregulated DASMs, the variation in a small amount of downregulated DASMs was less obvious during the entire five-year growth period. This variation trend based on interannual DASMs is consistent with that from the comparative analysis on the total flavonoid content and total saponin content in five-year-old *P. cyrtonema* rhizomes. Accordingly, the finding indicates an increasing accumulation trend in functional components with progressing growth years. The most active interannual accumulation occurred from the three- to four-year growth period, which suggests that the four-year-old *P. cyrtonema* rhizomes had the most superior quality compared with rhizomes of other ages.

Figure 5B shows that abundant up- and downregulated (233, 20.42% and 361, 31.64%) DASMs existed in the seasonal rhizomes. Among them, more flavonoids, phenolic acids, and alkaloids were downregulated, while more steroids were upregulated (Figure 6B). This result indicates that a drastic seasonal variation in the composition and abundance of secondary metabolites from *P. cyrtonema* rhizomes occurred during the spring–autumn growth period. This variation based on seasonal DASMs is consistent with that from the comparative analysis on the total flavonoid content and total saponin content in the rhizomes harvested in two seasons. Accordingly, the finding suggests that the *P. cyrtonema* rhizomes harvested in spring and autumn had unequal quality due to their significant differences in composition and content of functional components. Specifically, the rhizomes from spring contained more flavonoids, phenolic acids, and alkaloids, while the rhizomes from autumn contained more steroids.

### 2.5. Annotation and Functional Classification of Secondary Metabolites in P. cyrtonema Rhizomes

**Figure 7:**
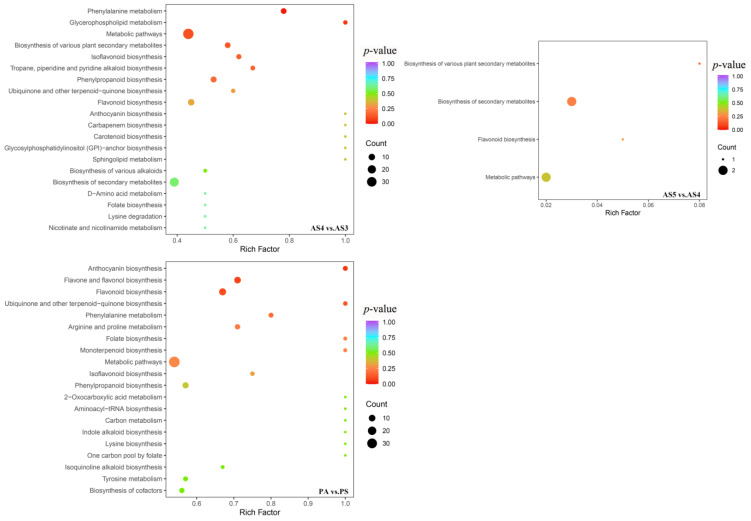
The top 20 significantly enriched KEGG pathways identified from AS4 vs. AS3, AS5 vs. AS4, and PS vs. PA. The color of dots in plots refers to higher or lower *p*-values, with bluer shades representing higher and redder shades representing lower. The size of a dot represents more or fewer secondary metabolites, with larger dots representing more and smaller dots representing fewer.

Enrichment analysis on KEGG pathways was performed on all identified secondary metabolites to determine which pathways associated with secondary metabolism are the most enriched with DASMs. The analysis was conducted between age sections of *P. cyrtonema* rhizome and between rhizomes harvested in spring and autumn. A total of 129 secondary metabolites from AS2 vs. AS1, 130 from AS3 vs. AS2, 136 from AS4 vs. AS3, and 136 from AS5 vs. AS4 were annotated to 10, 19, 30, and 4 KEGG pathways, respectively. Among all the annotated metabolites, nine from AS2 vs. AS1, twenty-six from AS3 vs. AS2, fifty-three from AS4 vs. AS3, and two from AS5 vs. AS4 were DASMs. Therefore, these DASMs were significantly enriched in these pathways associated with secondary metabolism. The most active interannual accumulation of functional components occurred during the three- to four-year growth period, and their decreased accumulation occurred after the four-year growth period. Thus, the enrichment analysis was focused on AS4 vs. AS3 and AS5 vs. AS4. The top 20 significantly enriched KEGG pathways in the two comparison groups are presented in Figure 7.

Among the 30 significantly enriched pathways identified from AS4 vs. AS3, most of them were related to the biosynthesis of various secondary metabolites and the metabolisms of some amino acids, including isoflavonoids (ko00943), flavonoids (ko00941), alkaloids (such as tropane, piperidine, and pyridine alkaloids, ko00960; isoquinoline alkaloids, ko00950), phenylpropanoids (ko00940), and terpenoids (ko00130), as well as phenylalanine metabolism (ko00360), D-Amino acid metabolism (ko00470), tyrosine (ko00350), and lysine degradation (ko00310). Meanwhile, most of DASMs enriched in these pathways were upregulated (Table S2). However, among AS5 vs. AS4, only four pathways related to the biosynthesis of secondary metabolites and flavonoids (ko01100, ko01110, ko00999, and ko00941) were identified as significantly enriched KEGG pathways, and only two DASMs were enriched in these pathways (Table S3). Therefore, these pathways related to the biosynthesis of secondary metabolites, especially flavonoid and alkaloid compounds, were significantly changed during the growth period from three to four years. This change was strongly associated with the increased accumulation of functional components during this period. However, these nearly unchanged pathways after the four-year growth period were also strongly associated with the slowing down accumulation of functional components during the growth period.

A total of 123 secondary metabolites from PA vs. PS were annotated to 33 KEGG pathways. Among them, nearly all the 62 DASMs that significantly enriched these pathways were downregulated. The top 20 significantly enriched KEGG pathways in this comparison group are presented in Figure 7. Among the 33 significantly enriched pathways, the biosynthesis of flavonoid and alkaloid compounds were the two dominating pathways, including flavonoids (such as anthocyanin, ko00942; ko00941), flavones and flavonols (ko00944), isoflavonoids (ko00943), indole alkaloids (ko00901), isoquinoline alkaloids (ko00950), and tropane, piperidine, and pyridine alkaloids (ko00960). In addition, some significantly enriched pathways were related to the biosynthesis of amino acids (ko01230) and the metabolisms of amino acids, such as phenylalanine (ko00360), arginine and proline (ko00330), lysine (ko00300), and tyrosine (ko00350) (Table S4). Therefore, these pathways related to the biosynthesis of secondary metabolites, especially flavonoid and alkaloid compounds, were extremely significantly changed from spring to autumn. This significant change was strongly associated with the decreased accumulation of functional components (except steroids) during this period. Meanwhile, these significantly enriched pathways related to the metabolisms of many kinds of amino acids suggest that significant changes in the chemical synthesis or degradation of amino acids occurred during the growth period from four to five years and also from spring to autumn.

In summary, KEGG enrichment analysis supports the results obtained from DASMs, which further explains the interannual and seasonal variations in functional components in *P. cyrtonema* rhizomes from the global metabolic pathways. These findings suggest that variable intracellular metabolic pathways, such as variations in the biosynthesis of secondary metabolites and amino acids during different growth periods, might be adaptive mechanisms for perennial *P. cyrtonema* to survive stress conditions over five years.

## 3. Discussion

Most functional components in medicinal plants are secondary metabolites, and their production depends greatly on the ecological stress conditions. Thus, plant secondary metabolism can be viewed as a behavior that contributes to adaptation and survival in response to environmental stress during the lifetime of the plant [14]. With regard to the mechanism involved in the induction of secondary metabolites, four previous hypotheses including (Carbon/Nutrient Balance), (Growth/Differentiation Balance), (Optimum Defense), and (Resource Availability) were proposed to explain the variation in secondary metabolites; they consistently concluded that environmental stress induced the increase in secondary metabolites [15]. For the purpose of harvesting medicinal materials with superior quality, some TCMs, such as *P. cyrtonema*, *Tetrastigma hemsleyanum*, *Paris polyphylla*, *Bletilla striata*, and *Panax notoginseng*, are usually planted under *P. edulis* forest with high canopy density (>0.7) and a cool and low-light growth environment, which is called “stress cultivation.” *P. cyrtonema*, as a perennial medicinal plant, has formed a set of mechanisms involved in morphological change, ecological adaptation, and physiological and biochemical responses to resist against various stress situations for surviving over many years. The interannual variation in secondary metabolites from *P. cyrtonema* rhizomes might be an adaptive physiological and biochemical mechanism acting to live under *P. edulis* forest over five years.

Considering the obvious differences in the secondary metabolites in the root and rhizome TCMs under different growth periods, determining suitable growth period and season for harvesting roots or rhizomes is important to ensure their superior quality and clinical efficacy, as indicated by the study on the accumulation of secondary metabolites in *A. sinensis* [16]. However, the increasing growth years were not correspondingly associated with the rising content of active components, as demonstrated by some studies on saponins in certain TCMs including *Polygala tenuifolia* and *Bupleurum chinenes* [17] and on baicalin, wogonoside, baicalein, and wogonin in the TCM *Scutellariae Radix* [18]. With the continuous growth from one to five years, the content of active ingredients in the stem of *Nauclea officinalis* increased continuously; however, the increasing rate began to slow down after reaching five years [19]. As a rhizome TCM, the DASMs in *P. cyrtonema* rhizomes during the growth period from three to four years reached 422, which was more than the total quantity of those (355) in rhizomes during the three periods including from one to two years, two to three years, and four to five years. This finding suggests that the fastest growth rate and the most active metabolism of functional components occurred during the growth period from three to four years. Subsequently, the metabolism slowed down after the four-year growth period. With the increasing growth years, perennial herbaceous plants will adopt a series of self-protection strategies to improve the probability of survival and reproduction success, such as slowing down growth rate and metabolic velocity and changing life history [20]. Unlike annuals transferring all nutrients into seeds, perennials store nutrients in manners that prolong their lifespans. The accumulation of storage compounds in perennating organs and the remobilization of these reserves occurs during the early stages of reactivation of growth [21]. Rhizome senescence of medicinal plant occurs in parallel with the progression of growth years; with the obvious rhizome senescence after the four-year growth period, the slowdown trend in the accumulation of secondary metabolites in *P. cyrtonema* rhizomes might be attributed to nutrient delivery from the old age sections to the young age sections during the budding time, which led to the decreased content of secondary metabolites in the old age sections. Overall, the variation trend of functional components in rhizomes might be an adaptive mechanism of perennial *P. cyrtonema* to cope with long-term environmental stress and survive under P. edulis forest, which also resulted in the superior quality of the four-year-old *P. cyrtonema* rhizomes for more types and the higher abundance of functional components contained in them.

Perennial herbs in seasonal climates need to optimize their carbon balance by adjusting their active season length, which is determined by spring growth and autumn senescence, to avoid risks of tissue loss under adverse conditions [22]. The biosynthetic pathways responsible for producing secondary metabolites undergo modulation during various growing seasons, which leads to either an increase or a decrease in the content of secondary metabolites [23]. Previous studies on some TCMs reflected the seasonal variations in functional components including saponins in *P. tenuifolia*, *Bupleurum chinense*, *Achyranthes bidentata*, and *Dioscorea pseudojaponica* [17]; rosmarinic acid, salvianolic acid B, crytotanshinone, and tanshinone ⅡA in *Salvia miltiorrhizae* [24]; taxoid, flavone, and polysaccharide in Needles of *Taxus wallichiana* var. mairei [25]; polysaccharides and total saponins in *P. cyrtonema* [8,9,10]; and isovinciside lactone in *N. officinalis* [19]. Chinese Pharmacopoeia (2015 edition) stipulates that spring or autumn is the suitable period for harvesting *P. cyrtonema* rhizomes, but their difference in functional components lacks data support. Our present study indicated that the rhizomes harvested in the two seasons have unequal quality due to their significant differences in composition and abundance of functional components. This finding suggests that the continuously changing climatic factors from spring to autumn greatly impact the metabolisms of secondary metabolites in *P. cyrtonema* cultivated under P. edulis forest, which results in significant changes during this period. Similar works concluded that the influence of season on chemical composition and thus biological activity can be attributed to climatic changes such as sunshine, temperature, soil humidity, and rainfall, as well as different stages of plant metabolism [26,27].

Quality markers (Q-markers hereinafter) that exist in the raw materials and products of TCM can be used as indicators for the quality control of TCM to embody their safety and effectiveness [28]. In recent years, the concept of Q-markers has significantly promoted the development of TCM [29]. In terms of the rhizomes of *Polygonatum* plants, besides *P. sibiricum* polysaccharide, flavonoids and saponins were preliminarily selected as Q-markers, which provided insights into their quality control and clinical use [30]. Flavonoids and their diglycosides, as Q-markers, are regarded as important functional components in *Polygonatum* plants, and their composition and yield in rhizomes are important quality indices of medicinal materials [31]. Although the content of total flavonoids showed a downward trend during the growth period from spring to autumn, various types of flavonoids in the *P. cyrtonema* rhizomes presented diversified variation characteristics including more newly formed than reduced, majority of upregulated, and minority of downregulated flavonoids (Table 2 and Table 3). Meanwhile, eight downregulated and two reduced homoisoflavonoids, as the characteristic components, were also included in these differentially accumulated flavonoids, as shown in Figure 4. This finding suggests that changing climatic factors from spring to autumn might impose considerable effects on the composition or decomposition of different flavonoids with similar structure, which leads to their multiple variations. The significantly seasonal variation trends of flavonoids might be mainly attributed to the long lighting time and sufficient rainwater that occurred in spring in Southeast China. Spring is the most active period for photosynthesis, and it promotes photosynthesis-dependent biosynthesis of flavonoids. Moreover, the spectral characteristics of *Phyllostachys pubescens* might be an equally important influencing factor resulting in the changes in flavonoids due to its significant seasonal fluctuations from spring to autumn [32]. With regard to the role of flavonoids against light stress, an important finding that the modulation in four flavonoids’ (kaempferol, quercetin, flavanol disaccharide I, and flavanol disaccharide II) content in cucumber was highly dependent on growing light spectra supported a spectral-dependent manner for the role of flavonoids in the regulation of light stress response [33,34]. Thus, the changed environmental factors from spring to autumn greatly influenced the metabolism of flavonoids, which led to their multiple variations. These phenomena were presented as a large quantity of newly formed and reduced metabolites. Overall, the decreased accumulation on flavonoids in autumn is a main seasonal variation, which did not result in better quality for the *P. cyrtonema* rhizomes harvested in this season.

Saponins in *Polygonatum* plants, as Q-markers, are rich and have various medicinal effects, such as anti-inflammatory, antiviral, insecticidal, and anticancer actions [35]. The content of total saponins in the *P. cyrtonema* rhizomes harvested in autumn was significantly higher than those in spring. Among them, 31 steroidal saponins showed increased accumulation, and 41 were newly formed. Meanwhile, diosgenin [C_27_H_42_O_3_], which was preliminarily selected as a Q-marker in *Polygonatum* plants, was a newly formed saponin in autumn. However, the abundance of eleven steroidal saponins in autumn were significantly decreased; among them, four were reduced to undetectable levels (Table 3 and Table S1). Overall, the increased accumulation on steroidal saponins in autumn was a main seasonal variation, which resulted in better quality of the rhizomes harvested in this season. Sunshine duration and intensity, light quality, environmental temperature, and annual rainfall were the dominating climatic factors to affect the accumulation of saponins in medicinal plants, which were proven by the studies on *Panax quinquefolius* (American ginseng) and *Panax notoginseng* (Chinese ginseng) [17,36]. Apart from light level, red and far red light and their ratio also significantly affected the ginsenoside content in two-year-old American ginseng roots collected in September [37]. In the *P. edulis* forest in Southeast China, the seasonal average air temperature ranged from 21 °C in spring to 32 °C in summer and then to 23 °C in autumn. Meanwhile, during the seasonal transitioning from spring to summer and then to autumn, sunshine duration and intensity and light spectra and quality in the *P. edulis* forest dynamically changed over time. Therefore, besides their own physiological status as an important internal factor leading to the increased accumulation of saponins in *P. cyrtonema* rhizomes, the significant seasonal variation might be due to the dynamic change in environmental factors including the dominating sunshine and light status, along with air temperature and rainfall in the *P. edulis* forest.

Our study provided evidence on the manner by which secondary metabolites show diversified and changeable responses toward stress conditions over five years and seasonal fluctuations from spring to autumn. It revealed that the most active interannual accumulation occurred during the three- to four-year growth period. However, future work is needed to understand the harvesting period in relation to bioactivity based on biossay of *P. cyrtonema* rhizome extracts and to comprehensively investigate what happened at a molecular levels using transcriptomics. This analysis would help understand the accumulation mechanism of functional components as a whole and specifically identify key genes responsible for the biosynthesis of flavonoids and steroidal saponins. In addition, KEGG enrichment analysis suggests that seasonal fluctuations greatly affected the biosynthesis and metabolisms of amino acids. Therefore, further study on seasonal variations in amino acids might be an interesting direction for future works, which will be expected to provide metabolic data for comparing the nutritional values of *P. cyrtonema* rhizomes harvested in spring and autumn. The quality of medicinal materials directly affects their chemical composition and clinical efficacy, which depends on cultivation mode under environmental stress and variety of TCM. To obtain high-quality medicinal materials made from *P. cyrtonema* rhizomes, we propose an idea of “quality breeding of *Polygonatum* plants,” which means selecting and breeding superior *Polygonatum* varieties with high content of functional components in the future work. Finally, with the increasing development of the TCM industry in China, an associative network among variety–environment–quality–metabolite–efficacy should be established for a standardized cultivation–harvesting processing system and a scientific quality evaluation method for the medicinal rhizomes.

## 4. Materials and Methods

### 4.1. Plant Material Collection and Pretreatment

**Figure 8:**
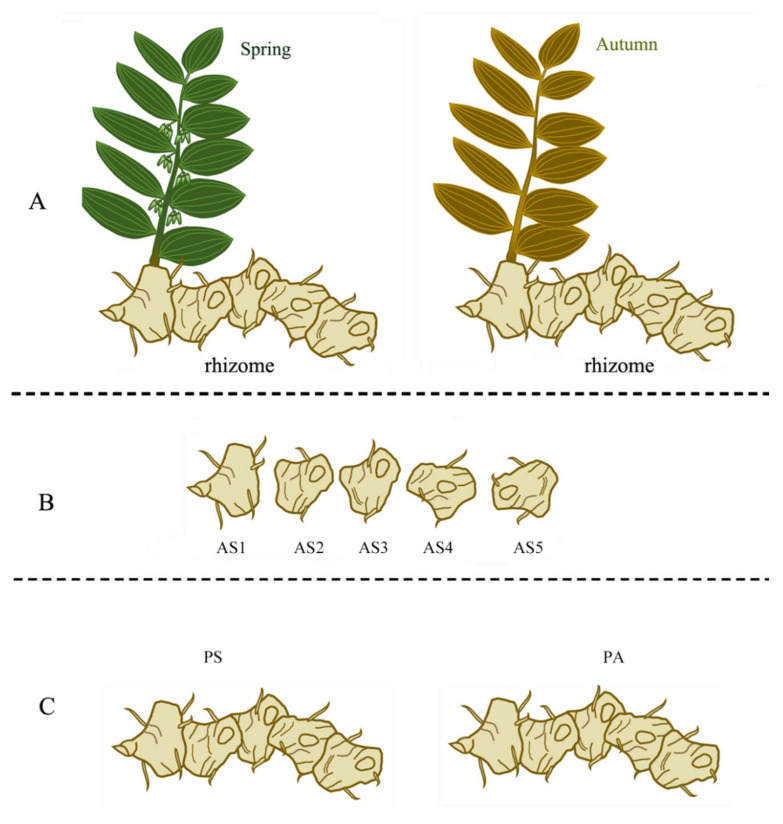
Five-year-old *P. cyrtonema* rhizomes used in this study. (**A**), *P. cyrtonema* plant; (**B**), the age sections of rhizome used for interannual variation analysis; (**C**), the entire rhizomes harvested in spring and autumn used for seasonal variation analysis. The letters AS1, AS2, AS3, AS4, and AS5 refer to the one-, two-, three-, four-, and five-year-old age sections, respectively; The letters PS and PA refer to the rhizomes harvested in spring and autumn, respectively.

During the period from April to October 2023, 15 fresh, healthy, five-year-old *P. cyrtonema* Hua. plants growing under *Phyllostachys edulis* forest (canopy density > 0.7) were collected from plantations in Quzhou (28°37′ N, 118°49′ E; altitude 446 m), Zhejiang Province (Southeast China). The rhizomes of these plants were used as test materials in this study (Figure 8A).

For interannual variation analysis, the *P. cyrtonema* rhizomes growing in spring (April) were harvested from nine plants and cleaned with deionized water. Then, one-, two-, three-, four-, and five-year-old age sections of rhizome were obtained from the entire rhizomes by cutting them with a scalpel (Figure 8B). Three age sections with the same year from three *P. cyrtonema* plants were mixed as a biological replicate. Thus, the entire rhizomes were divided uniformly into five interannual groups including one-year-old age sections (AS1 group) with three replicates (AS1a, AS1b, AS1c), two-year-old age sections (AS2 group) with three replicates (AS2a, AS2b, AS2c), three-year-old age sections (AS3 group) with three replicates (AS3a, AS3b, AS3c), four-year-old age sections (AS4 group) with three replicates (AS4a, AS4b, AS4c), and five-year-old age sections (AS5 group) with three replicates (AS5a, AS5b, AS5c). All fifteen age section samples in five groups were further cut into slices. Then, two-thirds of each sample was used to determine the total saponin content and total flavonoid content, while one-third was quickly frozen in liquid nitrogen Finally, the samples were stored at −80 °C until further metabolomics analysis.

For seasonal variation analysis, *P. cyrtonema* rhizomes were harvested from three plants growing in spring (April) and from three plants growing in autumn (October). These rhizomes were cleaned with deionized water (Figure 8C). The six rhizome samples were divided uniformly into two seasonal groups: one as spring rhizomes (PS group) with three replicates (PSa, PSb, PSc) and the other as autumn rhizomes (PA group) with three replicates (PAa, PAb, PAc). All these rhizome samples in two groups were further cut into slices. Thereafter, two-thirds of each sample was used to determine the total saponin content and total flavonoid content, while one-third was quickly frozen in liquid nitrogen. Finally, the samples were stored at −80 °C until further metabolomic analysis.

### 4.2. Determination of Total Flavonoid Content

The fresh *P. cyrtonema* rhizome samples were dried in an oven at 60 °C for 8 h, and the dried samples were grinded into fine powder. Two grams of rhizome powder were extracted with 32 mL of 90% ethanol. Forty milligrams of cellulase were dissolved in 400 μL of 0.1 M citric acid–sodium citrate buffer (pH 4.8), activated in a 40 °C water bath for 30 min, and then supplemented to the ethanol extract of rhizome powder. The extract was sonicated at 50 °C for 20 min, inactivated in boiling water for 10 min, and then filtered through filter paper. A UV/Vis full-wavelength scanning was performed to determine the absorption peak of total flavonoid extract. The UV/Vis spectra showed the maximum wavelength was 510 nm for rutin as standard sample. Thus, one milliliter of total flavonoid extract from rhizome powder was used to measure the absorbance at 510 nm using the UV/Vis Spectrophotometer (UV-6100, Metash, Shanghai, China) following the procedure described by [38,39]. The total flavonoid content was calculated from a standard curve with the concentration of rutin as equivalents (y = 0.0119x + 0.0060; R^2^ = 0.9996). The results were expressed in micrograms per gram of dried rhizomes (μg/g). All analyses were performed in triplicate. All values of total flavonoid content were expressed as means ± SD. Statistical significance was analyzed by one-way ANOVA and Duncan’s tests by using SPSS Statistics software (version 19, IBM Corporation, Armonk, NY, 2009). Differences were considered statistically significant at *p* < 0.0001, *p* < 0.001, *p* < 0.01, and *p* < 0.05.

### 4.3. Determination of Total Saponin Content

The fresh *P. cyrtonema* rhizome samples were dried in an oven at 60 °C for 8 h and then grinded into fine powder. The extraction of total saponins in rhizome powder and the determination of their content were performed according to the procedures described by [40]. Specifically, a total of 0.5 g of sample powder was extracted with 20 milliliters of water-saturated butane under the conditions of ultrasonic power (210 W, 60 °C) for 30 min, followed by centrifugation at a rotational speed of 5000 rpm for 5 min to remove insoluble materials. The supernatant obtained was collected and diluted with 20 mg/mL of water-saturated butane. One milliliter of the extracting solution was then mixed with 0.2 milliliters of 5% vanillin-glacial acetic acid. After being dried with nitrogen gas, the mixture was left to stand for 5 min. Subsequently, 0.8 milliliters of perchloric acid was added in the dried extract under ice bath conditions, and the mixture was placed in a water bath at 60 °C for 15 min. After the reaction was completed, it was placed in the ice again, and 5 milliliters of glacial acetic acid was added in it. The mixture was then left to stand for another 5 min, and finally, the absorbance was measured at 550 nm using the UV/Visible Spectrophotometer (UV-6100, Metash, Shanghai, China), and total saponin content was calculated from a standard curve with the concentration of diosgenin as equivalents (y = 3.1249x + 0.0687; R^2^ = 0.9991). The results were expressed in milligrams per gram of dried rhizomes (mg/g). All analyses were performed in triplicate. All values of total saponin content were expressed as means ± SD. Statistical significance was analyzed by one-way ANOVA and Duncan’s tests by using SPSS Statistics software (version 19, IBM Corporation, Armonk, NY, 2009). Differences were considered statistically significant at *p* < 0.0001, *p* < 0.001, *p* < 0.01, and *p* < 0.05.

### 4.4. Sample Preparation for Metabolomics Analysis

Sample preparation and extraction followed the methods provided by Metware Biotechnology Co., Ltd. (Wuhan, China), which was consistent with the previously published method used by our laboratory [41]. The extractions of the AS1, AS2, AS3, AS4, and AS5 samples were performed in triplicate. Three QC samples were prepared to test the repeatability of extraction and detection, and they were made by mixing all of the AS1, AS2, AS3, AS4, and AS5 sample extracts together. Similarly, the extractions of the PS and PA samples were performed in triplicate, and three QC samples were prepared by mixing all of the PS and PA sample extracts together.

### 4.5. UPLC-MS/MS and ESI-Q TRAP-MS/MS

The extracts of all the rhizome samples were detected based on the ultra-performance liquid chromatography electrospray ionization tandem mass spectrometry system [UPLC-ESI-MS/MS, UPLC, ExionLC™ AD, https://sciex.com.cn (accessed on 20 December 2023)] and electrospray ionization-triple quadrupole-linear ion trap mass spectrometry system (ESI-Q TRAP-MS/MS). The UPLC analytical conditions and the ESI source operation parameters were described according to the previously published method used by our laboratory [42].

### 4.6. Metabolite Annotation

The mass spectrometry data obtained using targeted multiple reaction monitoring (MRM) were subjected to qualitative and quantitative analyses according to the previously published method used by our laboratory based on the Metware database (MWDB v2.0, Wuhan, China) [42]. This approach combines the advantages of nontargeted and targeted metabolomics by using high-resolution triple quadrupole mass spectrometry (QQQ–MS) with high sensitivity, high specificity, and excellent quantitation capabilities. During the qualitative analysis, nontargeted repeat signals were eliminated according to the information of spectra of metabolites matching the MWDB of secondary metabolites, as described by [43,44]. The chromatographic peak area, which represented the integral data of the metabolites, was derived and used to calculate their relative abundance [45,46].

### 4.7. Multivariate Statistical Analysis

Multivariate statistical analyses were performed according to the previously published method used by our laboratory [42]. Briefly, metabolite data were log_2_-transformed and underwent autoscaling before any statistical analysis. Metabolite data from the AS1, AS2, AS3, AS4, and AS5 samples and from the PS and PA samples were used for unsupervised principal component analysis (PCA), hierarchical clustering analysis (HCA), and orthogonal partial least squares discriminant analysis (OPLS-DA) by using the Metware Cloud [https://cloud.metware.cn (accessed on 10 January 2024)]. OPLS-DA analysis referred to a previously published method by [47].

For the two-group analysis, the differential accumulated secondary metabolites (DASMs) were detected by variable importance in project scores (VIP ≥ 1) and fold change ≥ 2 or fold change ≤ 0.5, with VIP values extracted from the OPLS-DA results. The DASMs were annotated in pathways by using the Kyoto Encyclopedia of Genes and Genomes (KEGG) database [http://www.kegg.jp/kegg/compound (accessed on 12 January 2024)]. Pathways with significantly regulated metabolites were further subjected to metabolite set enrichment analysis, and their significance was determined by hypergeometric test’s *p*-values.

## 5. Conclusions

Overall, the total flavonoid content and total saponin content in *P. cyrtonema* rhizomes had a significant increase in accumulation during the one- to four-year growth period. The total flavonoid content was significantly decreased, while the total saponin content was significantly increased when the rhizomes were harvested in autumn. The functional components in *P. cyrtonema* rhizomes had a significant increase in accumulation during the one- to four-year growth period and a significant decrease in accumulation during the four- to five-year growth period. The most active accumulation occurred during the three- to four-year growth period. Drastic variations in types and abundance of functional components occurred from spring to autumn. The significant interannual variation and drastic seasonal variation were strongly associated with the enrichment in some pathways related to the biosynthesis of secondary metabolites and the metabolisms of amino acids, especially for flavonoid, alkaloid, and amino acids like phenylalanine and tyrosine.

*P. cyrtonema* has wide medicinal properties (e.g., anti-aging, immune function regulation, control of blood glucose and blood lipids, memory improvement, and antitumor and antibacterial effects), which is attributed to the abundant functional components derived from its rhizome, such as polysaccharides and secondary metabolites (e.g., saponins, flavonoids, and alkaloids). The quality of rhizomes depends on the composition and content of functional components, which directly affect their clinical efficacy as a Chinese herbal medicine. These results obtained from this study reveal that the interannual and seasonal variations in functional components can significantly affect the quality of *P. cyrtonema* rhizomes. The four-year-old rhizomes had the most superior quality because of their higher content of functional components and much more newly formed components. The rhizomes harvested in spring or autumn had unequal quality due to their significant differences in composition and abundance of functional components. Specifically, the rhizomes from spring contained more flavonoids, alkaloids, and phenolic acids, while those from autumn comprised more steroids. This study provides value insights and theoretical guidance for determining an optimal cultivation period to obtain medicinal rhizomes with superior quality. It also offers a strategy for harvesting medicinal rhizomes in two different seasons to achieve unequal quality.

## Figures and Tables

**Table 1:** The detailed information of the top 20 differential accumulated secondary metabolites (DASMs) with maximum values of Log_2_FC.

Comparison Group	Differential Accumulated Secondary Metabolites//Log_2_FC
AS2 vs. AS1	**Flavonoids (17):** Tamarixetin-3-*O*-glucoside-7-*O*-rhamnoside//2.81; Sexangularetin-3-*O*-glucoside-7-*O*-rhamnoside//2.78; Luteolin-7-*O*-gentiobioside//2.49; Isorhamnetin-3-*O*-Glucoside//2.45; 6-Methoxykaempferol-3-*O*-glucoside//2.37; Isorhamnetin-3-*O*-neohesperidoside//2.33; Isorhamnetin-3-*O*-sophoroside-7-*O*-rhamnoside//2.3; 2’-Hydoxy-5-methoxygenistein-*O*-rhamnosyl-glucoside//2.27; Isorhamnetin-7-*O*-glucoside (Brassicin)//2.25; Kaempferol-3,7-*O*-diglucoside//2.22; Okanin-4’-(6’‘-*O*-acetyl)-glucoside//2.14; Apigenin-6-*C*-(2’‘-xylosyl)-glucoside//2.13; Gallocatechin-(4*α*→8)-gallocatechin//2.11; Sesuvioside C//2.11; Scandione//2.11; kaempferol-3-feruloyldiglucoside-7-glucoside//2.05; Limocitrin 3-Glucoside//2.04 **Phenolic acids (1)**: Gentisic acid-[6’‘-(3-hydroxy-3-methylglutaryl)]-glucoside//2.57 **Terpenoids (1)**: Oxypaeoniflorin//3.78 **Others (1)**: Calyxanthone//3.74
AS3 vs. AS2	**Flavonoids (7)**: Phloretin-2’-*O*-(6’‘-*O*-xylosyl)-glucoside//3.57; (3R)-5,7-Dihydroxy-8-methyl-3-(2’,4’-dihydroxybenzyl)-chroman-4-one//-2.64; 3’-Hydroxy-4’-*O*-methylglabridin//-2.64; Naringenin-5-methyl ether//-2.59; Epiafzelechin//3.52; 5-hydroxy-7-methoxy-6,8-dimethyl-3-(2’-hydroxy-4’-methoxybenzyl)-chroman-4-one//2.78; Chrysoeriol-7-*O*-(6’‘-acetyl)glucoside//2.6 **Phenolic acids (2)**: Acetyl-2-*β*-*D*-glucopyranosyloxy-5-hydroxy-phenylacetic acid//2.42; Ethyl cinnamate//-3.37 **Alkaloids(1)**: 2-{5-hydroxy-2-[(6-hydroxy-7-methoxy-2-methyl-3,4-dihydro-1*H*-isoquinolin-1-yl)methyl]-4-methoxyphenoxy}-6-(hydroxymethyl)-oxane-3,4,5-triol//3.06 **Quinones (1)**: Aloesaponarin I//-4.03 **Terpenoids (4)**: Dehydrovomifoliol glucoside//2.44; Trijugin A//-4.07; Dendronobilin I-iso1//-3.22; Picroroside B//2.98 **Steroids (1)**: Protoaescigenin//-2.9 **Others (4)**: Machilusolide D//-3.46; 2-(2,4-dihydroxybenzyl)-6,8-dihydroxy-7-methyl-3,4-dihydronaphthalen-1(2*H*)-one//-2.4; Elaidolinolenic acid//-2.42; 3-Hydroxy-3,7,11-trimethyldodeca-1,6*E*,10-trien-9-yl isobutyrate(iso-1) //-2.43
AS4 vs. AS3	**Flavonoids (5)**: Hesperetin//4.79; Gossypetin-3-*O*-rutinoside//-5.59; (*E*)-3-(3’,4’-dihydroxybenzylidene)-5,7-dihydroxy-6,8-dimethyl-chroman-4-one//4.47; 7-Benzyloxy-5-hydroxy-3’,4’-methylenedioxyflavan//-4.56; Moracin Y//4.91 **Alkaloids (6)**: *N’*,*N’*‘,*N’*‘‘-*p*-Coumaroyl-cinnamoyl-caffeoyl spermidine//6.72; *N*-(3-hydroxy-4-methoxyphenethyl)-4-hydroxybutanamide//6.53; Broussonetinine B//5.09; Usaramine//4.85; Indole-3-carboxylic acid//4.56; Indole-5-carboxylic acid//4.44 **Steroids (4)**: Iso-diosgenin-Gal//6.32; Protoaescigenin//5.71; Spirost-5-ene-3,14-diol//5.33; Stauntoside R2//5.00 **Terpenoids (1)**: Chloranthalic acid//4.75 **Lignans and Coumarins (1)**: Desmethylagrimonolide-6-*O*-glucoside//4.67 **Others (3)**: 1-(9Z,12Z-Octadecadienoyl)-Sn-Glycero-3-Phosphocholine//-4.8; 1-18:3-LysoPC//-5.56; *O*-Phosphorylethanolamine//-4.75
AS5 vs. AS4	**Flavonoids (3)**: Syringetin-3-*O*-rutinoside-7-*O*-glucoside//1.68; Dactilin//-1.25; Petunidin-3,5-di-*O*-glucoside//-1.39 **Phenolic acids (2)**: 2-*O*-(3,4-dihydroxyphenylacetyl)-6-*O*-caffeoylglucoside//1.56; 4-Hydroxycinnamic acid p-hydroxyphenethylamine//-1.31 **Alkaloids (4)**: 7-Deoxynarciclasine glucoside//2.02; Neoverataline A//1.33; *Cis*-*N*-*p*-Coumaroyltyramine//-1.31; *p*-Coumaroyltyramine//-1.36 **Steroids (6)**: Diosgenin-Glc-Glc-Glc-diaccetyl Rha//2.12; Diosgenin-Glc-Glc-Glc-Glc-diaccetyl//1.44; 14-Dehydroxy-neoprazerigenin A-Glc-Glc-Glc-acetyl Glc//1.46; Gentrogenin-Glc-Glc-diacetyl Rha//1.88; 14-Dehydroxy-neoprazerigenin A-Glc-Glc-Glc-Xyl-Xyl//1.49; Iso-Pennogenin-Glc-Glc-Glc-acetyl Glc//1.27 **Quinones (1)**: 6-Methylaloe emodin//-2.68 **Terpenoids (3)**: Oleanolic acid//1.31; Jujuboside I//1.3; Oxypaeoniflorin//-2.07 **Others (1)**: 3’-Hydroxy-2-p-hydroxybenzyl-3,5-dimethoxybibenzene//-1.84
PA vs. PS	**Flavonoids (11)**: Myricetindiglucoside//6.41; 8-Demethylfarrerol//-5.85; 5,7-Dihydroxy-8-methyl-3-(2’,4’-dihydroxybenzyl)-chroman-4-one//-8.3; 5,7-Dihydroxy-6-methyl-3(*R*)-(2,4-dihydroxybenzyl)-chroman-4-one//-8.16; 7,3’-Dihydroxy-4’-methoxy-8-methylflavan//-6.01; 5,7-Dihydroxy-6-methyl-3-(2’,4’-dihydroxybenzyl)-chroman-4-one//8.07; Phloretin//-8.18; 5,7-Dihydroxy-3-(2’,4’-dihydroxybenzyl)-chroman-4-one//-6.72; Naringenin chalcone; 2’,4,4’,6’-Tetrahydroxychalcone//-6.16; 3-(4-Hydroxy)-5,7-dihydroxy-9-hydroxy-chroman-4-one//-6.57; Ophiopogonanone B//-7.33 **Steroids (6)**: Spirost-25(27)ene-2,3-diol-3-*O*-glucosyl(1→2)-galactoside//5.83; 3-Epidiosgenin-3-*O*-glucoside//6.36 Gentrogenin-Glc-Glc-Glc-Xyl-Xyl)//6.3; Trillin-6’-*O*-sophorotrioside//6.9; Pennogenin-Glc-Glc-Glc-Glc//6.05 3-Epineoruscogenin-Glc-Glc-Glc-glucoside//5.89 **Lignans and Coumarins (1)**: 3,4-Dihydro-4-(4’-hydroxyphenyl)-5,7-dihydroxycoumarin//-6.46 **Others (2)**: *β*-Hydroxypropiovanillone//-6.02; 6,8-Dihydroxy-2-(4-hydroxybenzyl)-5,7-dimethyl-3,4-dihydronaphthalen-1(2*H*)-one//-7.04

**Table 2:** The number of differential accumulated flavonoids and steroids in five comparison groups.

Comparison Groups	Flavonoids									Steroids	
	Flavone	Flavonol	Isoflavone	Other Flavonoid	Flavanol	Flavanonol	Chalcone	Anthocyanidin	Flavanone	Steroidal Saponin	Steroid
AS2 vs. AS1	13 (10↑/3↓)	33 (32↑/1↓)	6 (5↑/1↓)	8 (7↑/1↓)	1↑	1↑	1↑	0	0	8↑	0
AS3 vs. AS2	27 (16↑/11↓)	22 (21↑/1↓)	4 (1↑/3↓)	16 (7↑/9↓)	6↑	0	3↑	3↑	4 (2↑/2↓)	10↑	0
AS4 vs. AS3	37 (35↑/2↓)	13 (8↑/5↓)	9↑	45↑	7 (1↑/6↓)	1↑	7↑	4 (3↑/1↓)	12↑	25 (21↑/4↓)	5↑
AS5 vs. AS4	1↓	2↑	0	1↑	1↓	0	0	1↓	2 (1↑/1↓)	11↑	0
PA vs. PS	59 (37↑/22↓)	62 (9↑/53↓)	9 (1↑/8↓)	25 (1↑/24↓)	5 (1↑/4↓)	3↓	11 (↑1/10↓)	15 (6↑/9↓)	14↓	83 (72↑/11↓)	6↑

Note: ↑: upregulated differential accumulated secondary metabolites (DASMs); ↓: downregulated differential accumulated secondary metabolites (DASMs).

**Table 3:** The number of newly formed and reduced secondary metabolites identified from five comparison groups.

Comparison Groups	Newly Formed Secondary Metabolites	Reduced Secondary Metabolites
AS2 vs. AS1	Flavonoids: 1	Steroids: 1
	Alkaloids: 1	Lignans and Coumarins: 1
Total	2	2
AS3 vs. AS2	Flavonoids: 3	Flavonoids: 2
	Phenolic Acids: 3	Alkaloids: 1
	Alkaloids: 2	Quinones: 1
	Steroids: 2	
	Quinones: 2	
Total	12	4
AS4 vs. AS3	Flavonoids: 8	Alkaloids: 1
	Phenolic acids: 3	Terpenoids: 1
	Alkaloids: 5	Others: 1
	Steroids: 6	
	Terpenoids: 1	
	Lignans and Coumarins: 1	
	Others: 7	
Total	31	3
AS5 vs. AS4	Quinones: 1	Flavonoids: 2
	Lignans and Coumarins: 1	Others: 1
Total	2	3
PA vs. PS	Flavonoids: 45	Flavonoids: 77
	Phenolic Acids: 17	Phenolic Acids: 24
	Alkaloids: 2	Alkaloids: 6
	Steroids: 44 (Including 41 Steroidal Saponins)	Steroids: 3 (Steroidal Saponins)
	Terpenoids: 1	Terpenoids: 7
	Lignans and Coumarins: 8	Lignans and Coumarins: 8
	Others: 5	Quinones: 5
		Others: 18
Total	122	148

Note: In the AS2 vs. AS1 groups, newly formed secondary metabolite refers to its relative abundance in AS1 samples being undetectable, while reduced secondary metabolite refers to its relative abundance in AS2 samples being undetectable. Likewise, in the AS3 vs. AS2 groups, AS4 vs. AS3 groups, AS5 vs. AS4 groups, and PA vs. PS groups, newly formed secondary metabolite refers to its relative abundance in AS2, AS3, AS4, and PS samples being undetectable, while reduced secondary metabolite refers to its relative abundance in AS3, AS4, AS5 and PA samples being undetectable.

## Data Availability

The data are contained within the article and Supplementary Materials.

## Supplementary Materials

The following supporting information can be downloaded at https://www.mdpi.com/article/10.3390/plants13243459/s1. Figure S1: Principal component analysis (PCA) plot of secondary metabolites from fifteen age section samples (A) and six seasonal rhizome samples (B). The figure in brackets refers to the explained variance ratio. AS1, AS2, AS3, AS4, and AS5 represent the sample of one-, two-, three-, four-, and five-year-old age section of *P. cyrtonema* rhizomes, respectively. PS and PA represent the samples of spring rhizome and autumn rhizome, respectively. QC represents the mixture of *P. cyrtonema* rhizome sample extracts for repeatability test; Figure S2: OPLS-DA score plots of the five pairwise comparisons. AS1, AS2, AS3, AS4, and AS5 represent the samples of one-, two-, three-, four-, and five-year-old age section of *P. cyrtonema* rhizomes, respectively. PS and PA represent the samples of spring rhizome and autumn rhizome, respectively; Table S1: Newly formed and reduced secondary metabolites in each comparison group; Table S2: Number of metabolites in the comparion of AS4 vs. AS3 groups annotated to KEGG pathways; Table S3: Number of metabolites in the comparion of AS5 vs. AS4 groups annotated to KEGG pathways; Table S4: Number of metabolites in the comparison of PA vs. PS groups annotated to KEGG pathways.
