# Supplementary figures and images for: The Effect of Seasonal and Annual Variation on the Quality of Polygonatum Cyrtonema Hua Rhizomes
Source: Plants (Basel). 2024 Dec 10;13(24):3459. doi: 10.3390/plants13243459 (PMC11676584; doi:10.3390/plants13243459)

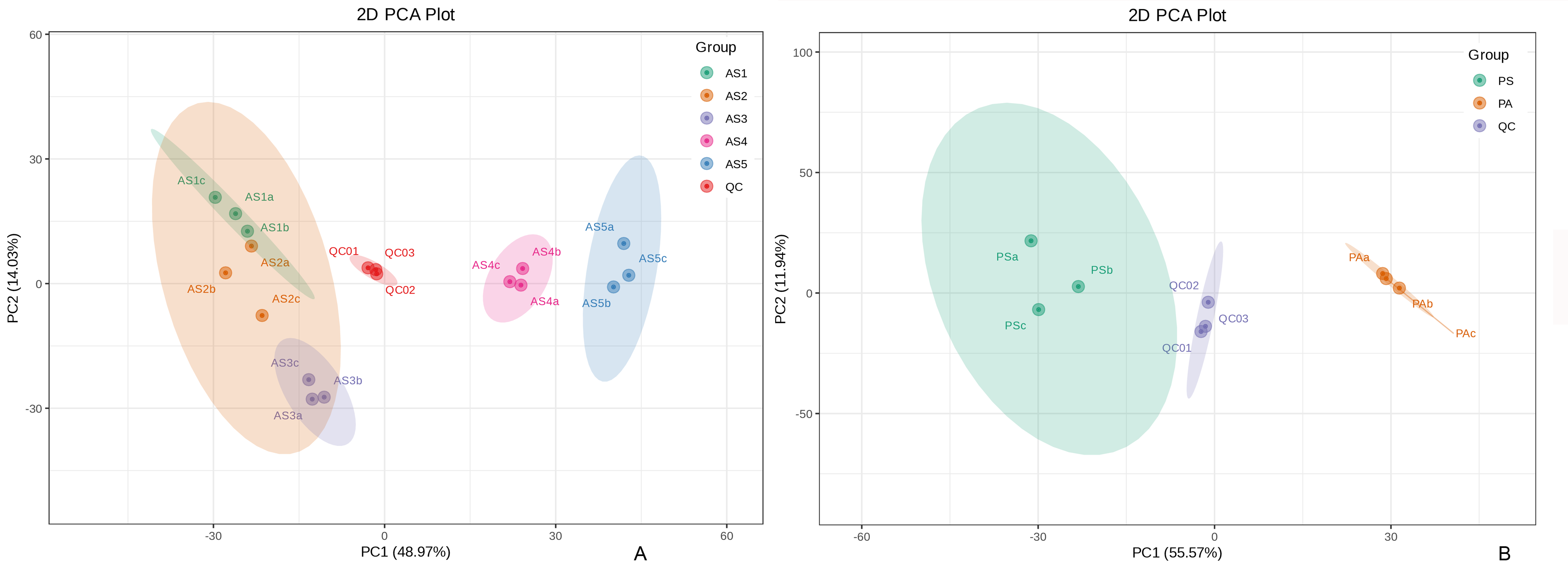

Supplement: Supplementary file 1 [file plants-13-03459-s001.zip › !Figure S1 Five year and PS-PA_PCA.tif]

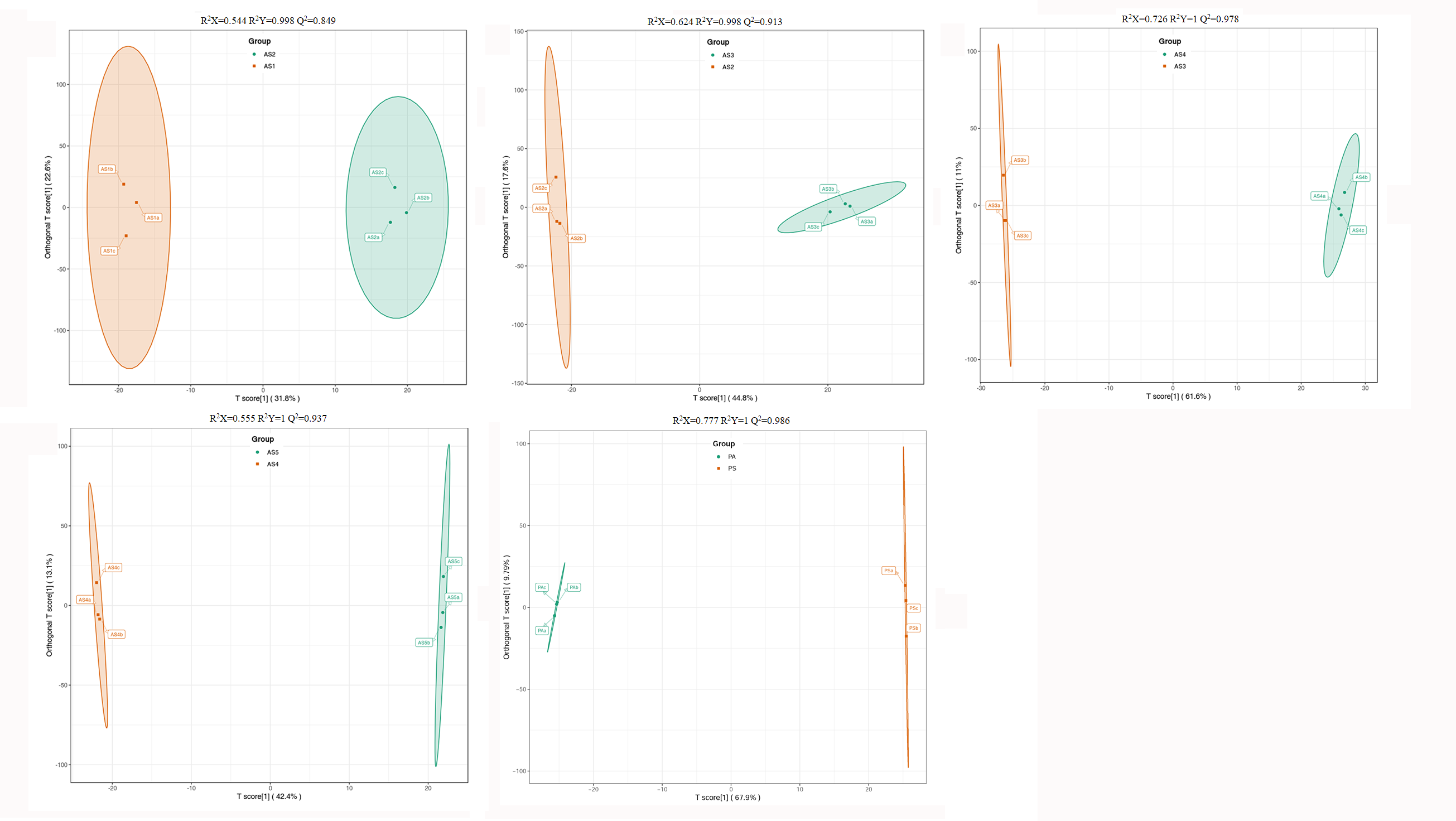

Supplement: Supplementary file 1 [file plants-13-03459-s001.zip › !Figure S2 OPLS-DA score plots of the five pairwise comparisons.tif]
